# Season, Transport Duration and Trailer Compartment Effects on Blood Stress Indicators in Pigs: Relationship to Environmental, Behavioral and Other Physiological Factors, and Pork Quality Traits

**DOI:** 10.3390/ani7020008

**Published:** 2017-02-08

**Authors:** Roberta Sommavilla, Luigi Faucitano, Harold Gonyou, Yolande Seddon, Renée Bergeron, Tina Widowski, Trever Crowe, Laurie Connor, Marina Bergoli Scheeren, Sébastien Goumon, Jennifer Brown

**Affiliations:** 1 Agriculture and Agri-Food Canada, Sherbrooke Research and Development Centre, Sherbrooke, QC J1M 0C8, Canada; robertasommavilla@gmail.com (R.S.); luigi.faucitano@arc.gc.ca (L.F.); marinabergoli@hotmail.com (M.B.S.); 2 Prairie Swine Centre, Saskatoon, SK S7H 5N9, Canada; hgonyou@shaw.ca (H.G.); yolande.seddon@usask.ca (Y.S.); 3 Department of Animal Biosciences, University of Guelph, Guelph, ON N1G 2W1, Canada; rbergero@uoguelph.ca (R.B.); twidowsk@uoguelph.ca (T.W.); 4 Department of Mechanical Engineering, University of Saskatchewan, Saskatoon, SK S7N 5A2, Canada; trever.crowe@usask.ca; 5 Department of Animal Science, University of Manitoba, Winnipeg, MB R3T 2N2, Canada; laurie.connor@umanitoba.ca; 6 Department of Ethology, Institute of Animal Science, Pratelstvi 815, 104 00 Prague-Uhrineves, Czech Republic; goumon.sebastien@vuzv.cz

**Keywords:** behavior, blood parameters, body temperature, heart rate, meat quality, pigs, season, stress response, transport time

## Abstract

**Simple Summary:**

Factors, such as ambient conditions, travel duration and vehicle design/compartment location have an impact on the welfare of pigs during transport, carcass bruises and meat quality. Based on this, we aimed to assess the effects of these factors on blood creatine kinase, lactate and cortisol concentrations in 384 pigs and assess their relationships with trailer temperature, and pigs’ heart rate and gastrointestinal tract temperature, behavior, carcass damage scores and meat quality. Although increased blood cortisol and creatine-kinase levels appear to indicate a physical stress condition in transported pigs, the weak to moderate correlations with environmental and other animal welfare indicators suggest that blood stress parameters can only be used as a complementary measurement in the assessment of the pigs’ response to transport stress.

**Abstract:**

The objective of this study was to assess the effects of the season, travel duration and trailer compartment location on blood creatine-kinase (CK), lactate and cortisol concentrations in 384 pigs and assess their relationships with trailer temperature, heart rate and gastrointestinal tract temperature (GTT), behavior, carcass damage scores and meat quality. Blood CK was greater in pigs transported in summer (*p* = 0.02), after 18 h transportation (*p* < 0.001) and in pigs located in C4, C5 and C10 (*p* = 0.002). In winter, the concentration of blood lactate was higher (*p* = 0.04) in pigs transported for 6 h in C5. Pigs located in C10 showed higher (*p* = 0.01) concentration of cortisol than those transported for 18h in C4 in summer. The highest correlations were between blood cortisol and GTT (*r* = 0.53; *p* < 0.001), and between blood CK and GTT (*r* = 0.41; *p* < 0.001), truck temperature (*r* = 0.42; *p* < 0.001), and pH_u_ in the *longissimus* muscle (*r* = 0.41; *p* < 0.001). In conclusion, although increased blood cortisol and CK levels appear to indicate a physical stress condition in transported pigs, the weak to moderate correlations with environmental and other animal welfare indicators suggest that blood stress parameters can only be used as a complementary measurement in the assessment of the pigs’ response to transport stress.

## 1. Introduction

Factors, such as ambient conditions, travel duration and vehicle design/compartment location have an impact on the welfare of pigs during transport, carcass bruises and meat quality [1,2]. Collecting blood at exsanguination is a common, non-invasive technique for the assessment of the pigs’ physiological response to these factors. Research has showed increased exsanguination blood CK and lactate levels in winter and summer, respectively [3,4], and in warmer trailer compartments [5]. Glucose, lactate and hematocrit levels are reported to increase in pigs transported for long duration [6,7], and blood cortisol and lactate concentrations to be greater after short journeys [8]. However, these results arise from studies where the effects of season, travel duration and compartment location were assessed separately. To our knowledge, the combined effects of these factors on exsanguination blood parameters have not been studied in pigs. Overall, correlations between blood stress parameters and other physiological indices, such as body temperature, behavior and meat quality range from none to moderate [9,10,11,12]. Nevertheless, correlations between exsanguination blood parameters, ambient and truck microclimate measures, and other physiological and behavioral response indicators to transport stress have not been calculated in one single study to validate their efficiency as a transport stress monitoring tool. 

The objective of this study was to assess the effects of the season, travel duration and truck compartment location on exsanguination blood parameters and calculate their correlations with internal truck temperature, heart rate and gastro-intestinal tract temperature, transport and post-transport behavior, carcass damage and meat quality.

## 2. Materials and Methods

All experimental procedures performed in this study were approved by the University of Saskatchewan’s Animal Research and Ethics Board and adhered to the Canadian Council on Animal Care guidelines for humane animal use [13].

### 2.1. Animals and Transport Procedures

The data reported in this experiment were collected during a larger study, whose results related to ambient and truck microclimate conditions, pigs’ gastrointestinal tract temperature, heart rate, behavior, carcass damage and meat quality have been reported in two companion papers [14,15]. Data reported in this paper were collected from the same animals described by Goumon et al. [14], and Scheeren et al. [15].

**Figure 1:**
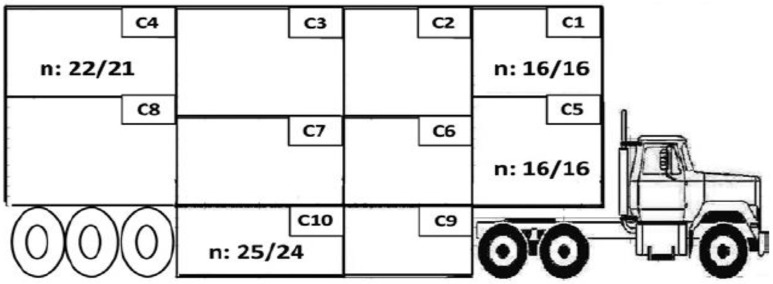
Compartment position inside the pot-belly trailer and distribution of pigs in the selected compartments in summer/winter.

In the overall study, a total of 5040 crossbred market-weight pigs (barrows and gilts) of the same genetics (Large White × Landrace sows and Duroc boars) and fed the same diet during finishing were transported from two commercial finishing farms to a commercial slaughter plant in Manitoba during the summer (July) and winter (January to February). Pigs were randomly distributed among three transport duration treatments: 6 h, 12 h, or 18 h in both seasons. Four replicates of each journey were conducted during each season over four weeks (total of 96 groups). The average ambient temperatures throughout the transport were 22.9 ± 1.7 °C (12.5 to 40.1 °C) in summer and −14.3 ± 1.7 °C (−28.8 to 1.9 °C) in winter. All trucks used were pot-belly types (tri-axle, natural ventilation, air suspension), transporting 210 pigs on three decks, distributed into 10 compartments (Figure 1). The side panels were open 100% in the summer and 10% in the winter. The truck was bedded with 10 bags (20 kg/bag) of wood shavings in summer and 8 bags of shavings and 9 bags of straw (20 kg/bag) in winter.

A subsample of 384 sentinel pigs (128 per treatment, 16 barrows/trailer; body weight: 120.8 ± 0.4 kg) was randomly selected for the physiological study, including blood analysis, and meat quality assessment. Sentinel pigs were transported in 4 compartments (4 pigs/compartment), namely top front (C1), top back (C4), middle front (C5), and rear bottom (C10; Figure 1), which were shown to be the most detrimental locations to animal welfare and meat quality in previous studies using the same trailer model [3,16]. 

Pigs transported for 18 h were off feed for approximately 24 h before slaughter, whereas those transported for 6 and 12 h were off for approximately 20 h. Loads travelling to slaughter for 18, 12, and 6 h left the farm at respectively 1300, 1900, and 0100 h. Each load consisted of unmixed pigs from the same farm and transported on the same truck. The different loads were transported by three different drivers, who were randomly assigned to transport duration treatments each week. The average space allowance on the truck was 0.37 m^2^/100 kg in summer and 0.38 m^2^/100 kg in winter. Throughout the loading procedure, pigs were handled by barn staff and drivers using plastic boards. Drivers and barn staff used electric prods under exceptional circumstances, such as moving reluctant pigs. The different loads arrived at the slaughter facility and were unloaded at 30 min intervals in a random, predetermined order beginning at 0630 h. Pigs were unloaded from their respective compartments in the reverse order of loading and staff only used paddles (not prods). Pigs from C1 and C4 and from C5 and C10 were grouped (*n* = 38–41) at unloading and moved to holding pens in lairage, with space allowance of 1.1–1.2 m^2^/pig. Pigs were provided *ad libitum* access to water in long troughs and held in lairage for ~150 min. After lairage, pigs were driven in a single file to the slaughter point, electrically stunned (head-to-chest stunning) and exsanguinated in the prone position. 

### 2.2. Blood Metabolites

Blood samples were collected at exsanguination for the analysis of creatine kinase (CK), cortisol and lactate. Other sampling times were not possible due to concerns regarding interference with normal abattoir procedures and the additional stress this would impose on live pigs. At exsanguination, 10 mL of blood were collected from the 384 sentinel pigs in a tube (BD Vacutainers, VWR International Ltd., Montreal, QC, Canada) to extract serum for CK and cortisol. Serum samples were kept at room temperature (~23 °C) for 1 h before refrigeration at 4 °C. The following day, serum samples were centrifuged at 4 °C for 12 min at 1400× *g*, transferred to 1.5 mL Eppendorf tubes, and stored at −80 °C until analysis. For the lactate analysis, another 2 mL of blood were collected in a tube containing 3.0 mg of sodium fluoride and 6.0 mg of Na_2_EDTA solution and were immediately centrifuged at 4 °C for 12 min at 1400× *g*. Plasma was transferred into 1.5 mL Eppendorf tubes and stored at −80 °C until lactate determination. CK levels were measured using a commercial kit (Creatine Kinase-SL, Sukisui Diagnostics, Charlottetown, PE, Canada) and serum CK concentrations were determined with a spectrophotometer. The quantitative determination of serum cortisol was made using a commercial Elisa kit (Neogen Corp., Lexington, KY, USA) with a microplate reader and expressed as ng/mL. Lactate levels were measured using Lactate Assay Kit (Biomedical Research Service Center, University of Buffalo, Buffalo, NY, USA) and plasma lactate concentrations were determined with a microplate. The intra-assay CV was 4.61%, 3.21% and 3.98% for plasma lactate, serum CK and cortisol, respectively.

### 2.3. Other Physiological Measures

#### 2.3.1. Heart Rate

Heart rate was recorded using Polar^®^ heart rate monitors (Team Polar, Polar Electro Canada Inc., Quebec, QC, Canada) at 5-s intervals from loading to unloading and it was applied 4 h before loading, fitted around the pig’s chest. The belts and monitors were removed immediately after unloading in order to avoid further interference with handling operations within the abattoir. 

#### 2.3.2. Gastrointestinal Tract Temperature

Gastrointestinal tract temperature (GTT) was monitored every minute from loading to lairage using iButton data. For administration, each pig was snared and a heavy gauge metal “pig gag” was inserted between its jaws to hold them open. A balling gun was used to insert the data logger into the pig’s mouth, and the pig was released and monitored for 30 s to ensure that the logger had been swallowed. After slaughter, the viscera were removed from the processing line, and the loggers were recovered from the viscera by gross dissection. A temperature drop of more than 2 °C between recordings during lairage was considered indicative of drinking, and data after this time point were excluded from calculations.

### 2.4. Behavioral Observations

#### 2.4.1. On-Truck Behavior

During transportation, behavior was recorded using digital cameras (Pentax Optio W90 12.1 MP, Mississauga, ON, Canada) mounted on the side of 3 of the compartments of interest (C1, C4 and C5) and programmed to take pictures at 5-min intervals. The cameras were mounted in such a way as to maximize the view of the compartments. No cameras were installed in C10 due to the lower height of the compartment. The percentages of animals within view that were standing, sitting or lying was recorded from the departure until the arrival at the slaughterhouse. 

#### 2.4.2. Lairage Behavior

Behavior during lairage was recorded by direct observation for 90 min beginning when the pigs entered the pen. Scan sampling was used at 5-min intervals to determine the number of pigs sitting, lying and drinking. The latency to rest was also determined as the time to when at least 50% of the pigs were lying. 

### 2.5. Truck Microclimate Measures 

One data logger (High Resolution Thermochron iButton DS1921H, Maxim Integrated Products, Inc., Sunnyvale, CA, USA) was mounted on the ceiling of each selected compartment in a central location to provide information about the temperature and relative humidity inside the compartments. The loggers were programmed to collect data at 5-min intervals throughout transport. 

### 2.6. Carcass Damage and Meat Quality Measurements

Skin damage was assessed by the same trained technician on the day of slaughter in the cooler using the 5-point photographic scale (1 = none to 5 = severe; [17]). 

Meat quality measurements were taken at 24 h *post-mortem* in the *Longissimus* (LM), *Semimembranosus* (SM) and *Adductor* (AD) muscles. Muscle ultimate pH (pH_u_) was assessed using a pH meter (Oakton Instruments Model pH 100 Series, Nilis, IL, USA) by inserting the glass probe into LM, SM and AD muscles. Instrumental color (L*, a*, and b* values) was measured in the LM and SM muscles with a Minolta Chromameter (CR-300; Minolta Canada Inc., Mississauga, ON, Canada) equipped with a 25-mm aperture, 0° viewing angle and D65 illuminant, after exposing the muscle surface to a 45-min blooming time. Drip loss was measured in the LM and SM muscles using the modified EZ-drip loss method as described by Correa et al. [18]. 

### 2.7. Statistical Analyses 

Blood and ambient temperature statistical analyses were done using the MIXED procedure in SAS (v9.2, SAS, 2010, Cary, NC, USA [19]), with fixed effects of season, transport duration and trailer compartment, and their 2 and 3-way interactions. Weeks were used as random effect. Blood CK concentrations were log-transformed to achieve normality. Pearson correlations were calculated between blood data and meat quality traits. Spearman correlations were calculated between blood data and truck microclimate measures, behavior observations and physiological measures. A probability level of *p* < 0.05 was chosen as the limit for statistical significance in all tests.

## 3. Results

### 3.1. Blood Parameters

As shown in Table 1, serum CK concentration was greater in summer (*p* = 0.03), after 18 h transportation (*p* < 0.001) and in pigs transported in C4 and C10 (*p* < 0.05). There was no interaction between season, duration and compartment for this blood variable. 

**Figure 2:**
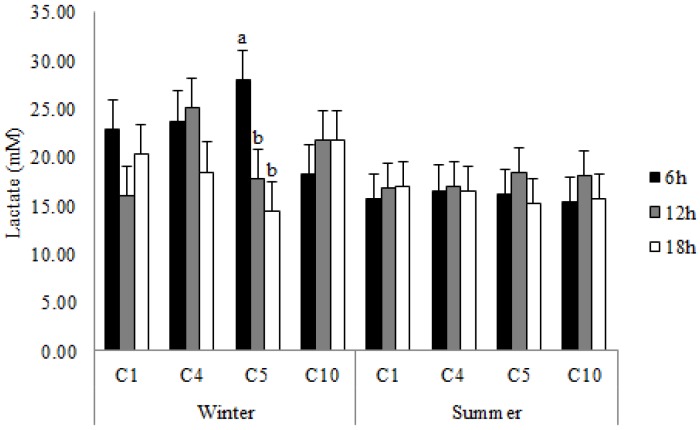
Blood lactate concentrations (LSM ± SEM) in pigs transported for 6, 12 and 18 h in the compartments C1, C4, C5 and C10 during winter and summer. ^a,b^ Least squares means without a common superscript differ (*p* < 0.05).

**Figure 3:**
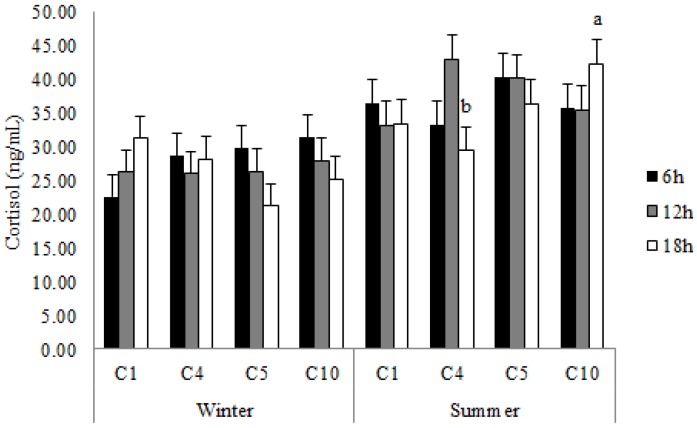
Blood cortisol value (LSM ± SEM) in pigs transported for 6, 12 and 18 h in the compartments C1, C4, C5 and C10 during winter and summer. ^a,b^ Least squares means without a common superscript differ (*p* < 0.05).

The interaction season x travel duration x compartment location influenced plasma lactate and serum cortisol concentrations in this study, with lactate levels being higher (*p* < 0.05) in pigs transported for 6 h in C5 in winter (Figure 2) and cortisol values being higher (*p* = 0.01) in pigs transported in C10 compared with those located in C4 in the 18-h journey in summer (Figure 3). 

### 3.2. Trailer Compartment Temperature and Relative Humidity Variation by Season and Compartment 

In this study, during winter, the temperature was lower (*p* < 0.001) in the top compartments (C1 and C4), while the temperature was higher (*p* < 0.001) in the bottom rear compartment (C10) during summer (Table 2). Compartment RH was also higher (*p* < 0.001) in the top compartments (C1 and C4) both in winter and summer. 

No effect of transport time was found on compartment temperature values in this study (*p* > 0.05).

### 3.3. Correlations between Blood Parameters and Microclimate, Other Physiological, Behavior and Meat Quality Variables, and Carcass Damage Scores

Descriptive statistics for heart rate, gastrointestinal tract temperature (GTT), transport and lairage behavior and pork quality values, and carcass damage scores are shown in Table 3.

Pearson correlation coefficients between blood parameters, heart rate, GTT, truck temperature, behavior, and meat quality characteristics are shown in Table 4. 

Serum CK concentration was positively correlated with serum cortisol level (*r* = 0.45; *p* < 0.001), carcass damage score (*r* = 0.29; *p* = 0.003), heart rate (*r* = 0.23; *p* = 0.02), GTT (*r* = 0.41; *p* < 0.001), trailer temperature (*r* = 0.42; *p* < 0.001) and pH_u_ value in the LM (*r* = 0.41; *p* < 0.001). Whereas, negative correlations were found between serum CK level and trailer RH (*r* = −0.36; *p* = 0.003), drip loss value in the LM and SM muscle (*r* = −0.23; *p* = 0.02 and *r* = −0.36; *p* < 0.001, respectively) and Minolta L* value in both muscles (*r* = −0.38; *p* < 0.001 and *r* = −0.25; *p* = 0.01, respectively). 

Blood cortisol level was positively correlated with GTT (*r* = 0.31; *p* = 0.002), trailer temperature (*r* = 0.53; *p* < 0.001) and lying behavior during transport (*r* = 0.41; *p* = 0.003). Whereas, it was negatively correlated with trailer RH (*r* = −0.49; *p* < 0.001) and standing behavior during transport (*r* = −0.41; *p* = 0.003). 

Blood lactate concentration was negatively correlated with GTT (*r* = −0.26; *p* = 0.009), trailer temperature (*r* = −0.38; *p* < 0.001) and pH_u_ value in the LM and SM muscle (*r* = −0.25; *p* = 0.02 and *r* = −0.32; *p* = 0.002, respectively). Whereas, it was positively correlated with trailer RH (*r* = 0.22; *p* = 0.01) and Minolta L* value in the LM (*r* = 0.21; *p* = 0.04). 

## 4. Discussion

### 4.1. Blood CK Variation

In this study, a significant effect of season was found for blood CK, with higher levels in summer, suggesting that heat stress played a significant role as shown by the significant, although moderate, positive correlations between this blood variable and pigs’ GTT and trailer internal temperature. Blood CK levels in this study rose significantly with increasing transport time, unlike the study by Chai et al. [20] which found no effect of transport times of 40 min, 3 h and 5 h on CK. This result is likely due to the much longer transport durations and more extreme temperature conditions examined in the current study. CK levels increase gradually in response to muscle exertion or damage, with peak levels found 6 h following injury [21]. This study also found compartment differences in CK, in contrast to the study by Correa et al. [4], which found no effect of location within the trailer on serum CK concentrations. Blood CK levels were generally higher in rear compartments (C4, C10) than in front (C1, C5), whereas Correa et al. [4] compared deck levels. The correlations of blood CK with muscle pHu and drip loss and colour lightness values confirm the role of this blood metabolite as a potential indicator of physical fatigue and muscle glycogen exhaustion, resulting in pork with DFD characteristics [3]. 

### 4.2. Blood Cortisol Variation

Blood cortisol concentrations were also influenced by the interaction season x transport time x compartment location in this study, with greater levels being found in summer, after 18 h travel time and in pigs located in the bottom rear compartment (C10). Based on the positive correlations with trailer temperature, which was greater in C10, pigs’ GTT and lying behavior during transport, the blood cortisol level increase may be interpreted as a response to heat stress which made pigs lie down more during transport as reported in the companion study by Goumon et al. [14]. Increased lying behavior is observed in pigs under warm ambient conditions with the objective of increasing the functional surface area for heat loss [22,23]. Greater cortisol concentrations after longer transport times (5 h vs. 40 min) have been also reported by Chai et al. [20] and may result from the additive effect of the 4 h longer feed withdrawal due to increasing demand for energy supply in these pigs [24].

### 4.3. Blood Lactate Variation

In previous research, lactate levels have been found to increase rapidly in response to acute stress, with a response time of 4 min [25]. In the current study, blood lactate increased in pigs transported in winter, but season effects were aggravated by the combined effects of shorter transport time and compartment location. The correlation between increased blood lactate level and decreased trailer temperature and GTT may explain the effects of winter on this blood variable in response to cold stress in this study. Greater lactate concentrations in exsanguination blood from pigs transported for a short time were also reported by Pérez et al. [8]. The effects of low temperatures and shorter travel time were accentuated in pigs located in the middle front compartment (C5) despite the fact that this compartment was the warmest during the winter trials in this study. Other studies found higher blood lactate concentrations during winter [4,26,27] and correlated with the longer latency to lie down in lairage and delayed recovery from transport in this season [4]. However, this interpretation cannot be applied for the results of this study as no significant correlation was found between blood lactate level at exsanguination and standing/lying behavior in lairage. Similarly to a number of studies [3,10,11,12], blood lactate increase contributed, although weakly, with *post-mortem* meat acidification resulting in paler pork. 

## 5. Conclusions

The results of this study suggest that pigs transported at elevated truck temperatures are likely to experience higher stress showed by the increase of blood cortisol and CK levels, combined with higher frequencies of lying during transport and drinking in lairage. These effects are exacerbated by the position of the animal in the trailer during transportation, meaning that the design of vehicles used for swine transportation in Canada should be improved to ensure more consistent comfort for all animals, regardless of their position in the truck, during travel. However, based on the weak to moderate correlations with environmental, other physiological and behavior indicators and meat quality traits, the stress parameters measured in blood collected at exsanguination in this study cannot be considered other than as a complementary measurement in the assessment of the physiological response to transport stress.

## Figures and Tables

**Table 1:** Variation in blood lactate, creatine kinase (CK) and cortisol concentrations by season (winter and summer), travel duration (6, 12 and 18 h) and trailer compartment location (C1, C4, C5 and C10).

	Season (S)			Duration (D)				Compartment (C)					*p* Value		
*n*	Winter 48	Summer 48	SEM	6 h 32	12 h 32	18 h 32	SEM	C1 24	C4 24	C5 24	C10 24	SEM	S	D	C
CK * (U/L)	2734 ^b^ (2054–3639)	4424 ^a^ (3324–5888)		2720 ^b^ (2212–3343)	3542 ^a^ (2881–4354)	4367 ^a^ (3552–5368)		2612 ^b^ (2087–3269)	4194 ^a^ (3351–5248)	3436 ^a,b^ (2745–4300)	3887 ^a^ (3106–3887)		0.03	<0.001	0.002
Lactate (mM)	20.72	16.53	1.89	19.86	18.86	17.43	1.48	18.12	19.58	18.32	18.48	1.54	0.17	0.14	0.66
Cortisol (ng/mL)	27.10 ^b^	36.60 ^a^	2.06	32.28	32.32	30.95	1.67	30.56	31.43	32.37	33.04	1.77	0.02	0.55	0.47

* The values in brackets are lower and upper values of 95% confidence interval. ^a,b^ Within a row, least squares means without a common superscript differ (*p* < 0.05).

**Table 2:** Least square means, SD, minimum and maximum values for temperature and relative humidity inside the selected trailer compartments (C1, C4, C5 and C10) during winter and summer.

Season		Temperature (°C)				Relative Humidity (%)			
		C1	C4	C5	C10	C1	C4	C5	C10
Winter	Mean	−0.9 ^b^	−1.6 ^b^	7.1 ^a^	6.1 ^a^	90.22 ^a^	92.29 ^a^	77.94 ^b^	79.82 ^b^
	SD	4.18	4.07	2.74	4.02	8.47	7.69	9.86	8.53
	Min.	−16.5	−14	−12.5	−10.5	81.74	83.82	64.87	65.72
	Max.	5.2	6.1	10.2	18.6	99.55	99.66	89.28	89.96
Summer	Mean	23.9 ^b,c^	23.1 ^c^	24.7 ^ab^	25.3 ^a^	72.96 ^a^	73.64 ^a^	68.04 ^b^	69.17 ^b^
	SD	3.53	3.35	3.08	3.13	8.64	8.37	7.51	8.30
	Min.	13.7	13.2	15.7	16.12	57.66	58.67	56.97	49.18
	Max.	38.16	36.7	35.2	34.6	84.18	85.02	79.5	76.69

^a,b,c^ Within a row, least squares means lacking a common superscript differ (*p* < 0.001).

**Table 3:** Descriptive statistics for heart rate, gastrointestinal tract temperature (GTT), transport and lairage behavior and pork quality values (as assessed in the *longissimus*, LM, *semimembranosus*, SM, and *adductor*, AD, muscles), and carcass damage scores.

Parameter	Mean	SD	Minimum	Maximum
Heart rate	119.23	16.94	84.10	173.33
GTT (°C)	39.26	0.46	38.18	41.17
Transport behaviors				
Standing (%)	32.33	21.57	4.18	85.76
Sitting (%)	17.74	6.92	1.05	33.19
Lying (%)	44.71	22.26	0	80.76
Lairage behaviors				
Standing (%)	2.20	1.91	0	10.37
Lying (%)	73.14	9.98	43.33	96.11
Drinking (%)	2.93	2.09	0.20	8.94
Carcass damage score ^1^	1.77	0.40	1.00	3.50
Meat quality measurements				
LM pH_u_	5.87	0.14	5.55	6.78
LM Minolta color L*	44.80	2.60	37.51	53.80
LM drip loss (%)	2.88	1.52	−2.03	10.72
SM pH_u_	5.84	0.15	5.37	6.46
SM Minolta color L*	43.77	3.18	34.10	51.61
SM drip loss (%)	3.23	1.42	−2.71	7.08
AD pH_u_	5.97	0.23	5.26	6.74

^1^ 1 = none to 5 = severe (MLC, 1985).

**Table 4:** Correlations (Pearson, *r* values) between blood parameters, heart rate, gastrointestinal tract temperature (GTT), trailer temperature (T°) and relative humidity (RH), transport and lairage behaviors, carcass damage score and meat quality characteristics as assessed in the *longissimus* (LM), *semimembranosus* (SM) and *adductor* (AD) muscles.

Parameter	CK	Cortisol	Lactate	Heart Rate	GTT	Standing (Transport)	Sitting (Transport)	Lying (Transport)	Standing (Lairage)	Lying (Lairage)	Drinking (Lairage)	Carcass Damage Score	Compartment T°	Compartment RH	LM pH_u_	LM Minolta Color L*	LM Drip Loss	SM pH_u_	SM Minolta Color L*	SM Drip Loss	AD pH_u_
*r*																					
*p*																					
*n* ^1^	96	96	96	95	96	48	48	48	96	96	96	96	88	88	84	96	96	84	94	94	84
CK ^2^	1	0.45	−0.06	0.23	0.41	−0.08	0.23	0.02	−0.15	−0.19	0.05	0.29	0.42	−0.36	0.41	−0.38	−0.23	0.08	−0.25	−0.36	0.013
	-	<0.001	0.57	0.02	<0.001	0.56	0.11	0.89	0.13	0.06	0.65	0.003	<0.001	0.003	0.0001	0.0001	0.02	0.48	0.01	<0.001	0.9
Cortisol		1	0.04	0.08	0.31	−0.41	0.21	0.41	−0.08	−0.07	−0.01	0.07	0.53	−0.49	−0.08	−0.04	0.08	−0.15	0.06	0.12	−0.12
		-	0.69	0.44	0.002	0.003	0.15	0.003	0.46	0.47	0.9	0.46	<0.001	<0.001	0.44	0.73	0.41	0.17	0.54	0.23	0.27
Lactate			1	−0.01	−0.26	−0.12	−0.01	0.12	0.04	0.07	−0.14	0.05	−0.38	0.22	−0.25	0.21	0.01	−0.32	0.25	−0.08	0.13
			-	0.91	0.009	0.43	0.95	0.43	0.68	0.48	0.16	0.61	<0.001	0.01	0.02	0.04	0.9	0.002	0.01	0.42	0.24

^1^ *n*: Unit was trailer compartment. For postures during transport (*n* = 48) two compartments were analysed per trailer, and the values were correlated against the corresponding blood and meat quality values for trailer compartment. ^2^ CK: creatine kinase.
