# Addendum: Sommavilla, R. et al. Season, Transport Duration and Trailer Compartment Effects on Blood Stress Indicators in Pigs: Relationship to Environmental, Behavioral and Other Physiological Factors, and Pork Quality Traits. *Animals* 2017, *7*, 8

**DOI:** 10.3390/ani7030013

**Published:** 2017-02-24

**Authors:** Roberta Sommavilla, Luigi Faucitano, Harold Gonyou, Yolande Seddon, Renée Bergeron, Tina Widowski, Trever Crowe, Laurie Connor, Marina Bergoli Scheeren, Sébastien Goumon, Jennifer Brown

**Affiliations:** 1 Agriculture and Agri-Food Canada, Sherbrooke Research and Development Centre, Sherbrooke, QC J1M 0C8, Canada; robertasommavilla@gmail.com (R.S.); luigi.faucitano@arc.gc.ca (L.F.); marinabergoli@hotmail.com (M.B.S.); 2 Prairie Swine Centre, Saskatoon, SK S7H 5N9, Canada; hgonyou@shaw.ca (H.G.); yolande.seddon@usask.ca (Y.S.); 3 Department of Animal Biosciences, University of Guelph, Guelph, ON N1G 2W1, Canada; rbergero@uoguelph.ca (R.B.); twidowsk@uoguelph.ca (T.W.); 4 Department of Mechanical Engineering, University of Saskatchewan, Saskatoon, SK S7N 5A2, Canada; trever.crowe@usask.ca; 5 Department of Animal Science, University of Manitoba, Winnipeg, MB R3T 2N2, Canada; laurie.connor@umanitoba.ca; 6 Department of Ethology, Institute of Animal Science, Pratelstvi 815, 104 00 Prague-Uhrineves, Czech Republic; goumon.sebastien@vuzv.cz

The authors would like to add that “Sébastien Goumon was supported by Grant No MZERO0716 from the Czech Ministry of Agriculture” in the Acknowledgement Section of their paper published in *Animals* [1]. 

The authors apologize for any inconvenience this change may cause. The changes do not affect the scientific results. The manuscript will be updated and the original will remain online on the article webpage, with a reference to this addendum.
